# Disease Intervention Specialist-Delivered Interventions and Other Partner Services for HIV and Sexually Transmitted Infections: A Systematic Review

**DOI:** 10.1016/j.amepre.2024.08.004

**Published:** 2024-08-12

**Authors:** Erika G. Martin, Arzana Myderrizi, Heeun Kim, Patrick Schumacher, Soyun Jeong, Thomas L. Gift, Angela B. Hutchinson, Kevin P. Delaney, Harrell W. Chesson

**Affiliations:** 1 Public Health Accreditation Board, Alexandria, Virginia;; 2 Department of Public Administration and Policy, Rockefeller College of Public Affairs and Policy, University at Albany, Albany, New York;; 3 Division of STD Prevention, National Center for HIV, Viral Hepatitis, STD, and TB Prevention, Centers for Disease Control and Prevention, Atlanta, Georgia;; 4 Division of HIV Prevention, National Center for HIV, Viral Hepatitis, STD, and TB Prevention, Centers for Disease Control and Prevention, Atlanta, Georgia

## Abstract

**Introduction:**

Disease intervention specialists (DIS) are critical for delivering partner services programs that provide partner notification, counseling, referral, and other services for HIV, sexually transmitted infections (STIs), and other infections. This systematic review of partner services and other DIS-delivered interventions for HIV and STIs was conducted to summarize the effectiveness of these programs and identify evidence gaps.

**Methods:**

A systematic literature review was conducted with a narrative synthesis. Articles were located using keyword searches in MEDLINE, Web of Science, CINAHL, and ProQuest through December 2022 and analyzed in 2023−2024. Included studies addressed an intervention of partner services or other DIS-delivered services for HIV or STIs; a United States setting; primary data collection; and an external comparison group or pre-post design.

**Results:**

A total of 1,915 unique records were screened for eligibility, with 30 studies included. Overall, DIS-delivered interventions improved clinical outcomes among index patients and population outcomes. Many studies focused on program process measures rather than population-level epidemiologic outcomes. All but one studies were scored as having low or medium strength of evidence.

**Conclusions:**

The evidence could be strengthened by establishing a streamlined set of core metrics, assessing impact using rigorous causal inference methodologies, linking program and clinical data systems, and supplementing impact evaluations with evidence on implementation strategies.

## INTRODUCTION

Disease intervention specialists (DIS) deliver partner services programs encompassing a wide range of activities for HIV and sexually transmitted infections (STIs) including field testing and treatment, expedited partner therapy, case treatment follow-up, partner notification, counseling, and referral services.^1,2^ They collect crucial data for disease surveillance. Additionally, they played a pivotal role in the COVID-19 response.^3^ Enhancing the DIS workforce is part of the federal strategy to strengthen outbreak response capacity.^4^

Past reviews have examined different aspects of DIS-delivered interventions for HIV and STIs.^5–9^ Other reviews focused on new technologies in HIV/STI partner services.^10,11^ Overall, there is evidence for a positive impact of DIS-delivered interventions. An updated systematic review is warranted considering evolutions in partner services, the DIS workforce, syndemic approaches, and epidemiological characteristics that influence outcomes such as test positivity among partners.

This review addressed the following questions: What is the program impact of partner services and other DIS-delivered interventions for HIV and STIs on clinical and epidemiological outcomes among index patients and their partners in the U.S.? How do outcomes vary across infections? What are the critical evidence gaps? For consistency with impact evaluations employing experimental or non-experimental designs such as pre-post or post-test comparisons between groups to assess program effectiveness,^12,13^ this review considered comparisons to no intervention, alternative interventions not delivered by DIS, or different modes of delivery.

## METHODS

This systematic literature review used a narrative synthesis of findings following National Academy of Medicine and Patient Centered Outcomes Research Institute (PCORI) methods^14,15^ and the Preferred Reporting Systems for Systematic Reviews and Meta-analyses (PRISMA) reporting guidelines.^16^ The protocol was registered with PROSPERO (CRD42022375961).^17^ The study did not require University at Albany institutional review board review because it was a literature review. Although the protocol encompassed DIS-delivered services for HIV, STIs, and tuberculosis, tuberculosis studies were excluded because analyses of the effectiveness of tuberculosis contact tracing were too dissimilar from HIV and STIs to allow for meaningful comparisons, and only 4 tuberculosis studies were found.^18–21^ A second protocol deviation was excluding conference abstracts because they provided insufficient information to extract study characteristics and assess the strength of evidence.

The keyword search was implemented in PubMed/MEDLINE, CINAHL, ProQuest Dissertations & Theses Global, and Web of Science. The medRxiv preprint server was not included because it was infeasible to implement a similar keyword search with terms and Boolean operators; additionally, studies were not peer-reviewed. The keywords were developed based on the study team’s subject matter expertise, pilot searching, and consultation with 2 reference librarians. After the initial search, a second search was conducted with additional keywords for “contact investigation” (a synonym for contact tracing) and “venue-based testing” (a DIS-delivered intervention). The keyword search was completed in December 2022 and supplemented by a hand search of references in included studies and relevant review articles. Analyses including the hand search were conducted during 2023−2024. The Appendix contains additional details.

Population, intervention, comparator, outcomes, timing, and setting (PICOTS) screening criteria were used by authors EM, AM, HK, PS, and SJ. Additionally, included studies met a criterion of primary analysis of empirical data, excluding literature reviews and modeling studies that did not present new empirical data to derive model parameters. The population criterion included persons recently diagnosed with or at risk for HIV or non-viral STIs and their partners. Medical and community-based providers and program staff were excluded. The intervention criterion covered partner services and other DIS-delivered interventions; specific activities included interviewing index patients to elicit partner information, partner notification, screening, vaccination against related infections, prevention counseling, presumptive treatment, expedited partner therapy, linkage or referral to medical care and other services, direct delivery or referrals for HIV pre-exposure prophylaxis, and venue-based screening. Interventions not delivered by DIS were excluded; for example, expedited partner therapy was only included if DIS-delivered. Data-to-care interventions for persons with HIV presumed to be out of care and social network interventions were excluded because they target individuals previously in care. The comparator criterion required a comparison of outcomes between populations that did and did not receive partner services or a pre-post comparison of outcomes before and after receiving partner services, including studies that compared different program delivery modalities. Studies with a comparator but no statistical test of significance were excluded. In terms of outcomes, all outcomes for index patients, partners, and the community along the continuum of clinical evaluation, counseling, diagnostic testing, and treatment were considered. Receipt of partner services was excluded as an outcome. Regarding timing, all studies were considered. The setting criterion was the United States due to variation in interventions and terminology in non-U.S. settings.

All studies identified in the keyword search were reviewed manually for eligibility in 2 phases. First, the titles and abstracts were screened based on the primary research and PICOTS criteria using hierarchical criteria (ordering: setting, primary research, intervention, comparator, population, and outcomes). At this stage, screeners (AM, HK, and PS) were flexible in progressing studies to the second stage if exclusion was unclear. Second, full-text articles were screened. Following the PCORI guidance that relaxes standards related to dual screening and data abstraction if fact-checking and quality control procedures are in place,^15^ the first stage screening was conducted by 1 author. Screeners were trained by double-coding at least 30 studies with the first author and flagged articles with unclear determinations for review by a second screener. In the first stage, 534 (33.3%) of the 1,605 journal articles were reviewed by a second screener. The lead author reviewed all ProQuest studies that were not removed by screeners for non-US setting or lacked relevance. In the second stage full-text review, all 138 studies were screened by 2 authors. Where decisions were unclear, one or more additional authors reviewed to resolve via consensus. For additional studies identified in the hand search of references of included articles from the keyword search, 2 screeners (AM, HK) reviewed references independently and verified each other’s decisions.

Reviewers (AM, HK, PS, and SJ) manually extracted the following into a standardized, pre-piloted form (see the Appendix): stated aims, intervention description, infections examined, study design, statistical analysis, comparator, data source and coverage, location, population, study sample, outcome measures, key findings, and reported limitations. One reviewer extracted information, with a second verifying the extraction. Questions were resolved through consensus and the lead author when needed. Additionally, all summaries were reviewed by the senior author. Only findings reporting a statistical test of significance (*p*-values or confidence intervals) were extracted. Anticipating heterogeneity across studies, no restrictions were placed on effect measures (e.g., risk ratios or mean differences). If there were multiple analyses, the highest-order analysis was extracted (e.g., multivariable regression results were extracted rather than bivariate results). Missing or unclear information was not confirmed with study investigators because it was infeasible to contact all authors.

Findings were summarized by distilling study characteristics and outcomes from the data extraction sheets into summary tables. A narrative synthesis summarized findings by infection, specific interventions, and components of the disease intervention cascade (interview, testing, treatment, and risk behaviors for index patients; partner identification, notification, testing, and treatment; and population-level epidemiological outcomes). Due to the diverse outcome measures, interventions, and infections, it was infeasible to perform a meta-analysis or conduct other quantitative syntheses.

For each study, the strength of evidence on program effectiveness was determined by considering holistically the JBI critical appraisal checklists,^22^ study design, and key limitations. The appraisal was specific to studies with a research design suitable for an impact evaluation. One reviewer completed the checklists, writing detailed comments assigning an overall strength of evidence score of “high,” “medium,” or “low” based on the checklist scores, study design, and study limitations (e.g., small sample sizes or nonequivalent groups with insufficient adjustment in multivariable analysis). A second reviewer verified scores and wrote additional comments regarding their own interpretations. All disagreements were resolved through consensus and discussion with the lead author (EM) when needed. See the Appendix for additional details.

It was infeasible to conduct a formal assessment of the certainty in the body of evidence for the effectiveness of partner services and other DIS-delivered interventions due to the variability of studies with respect to specific interventions, infections included, and study design. Instead, the key limitations and strength of evidence were described narratively.

## RESULTS

**Figure 1.**
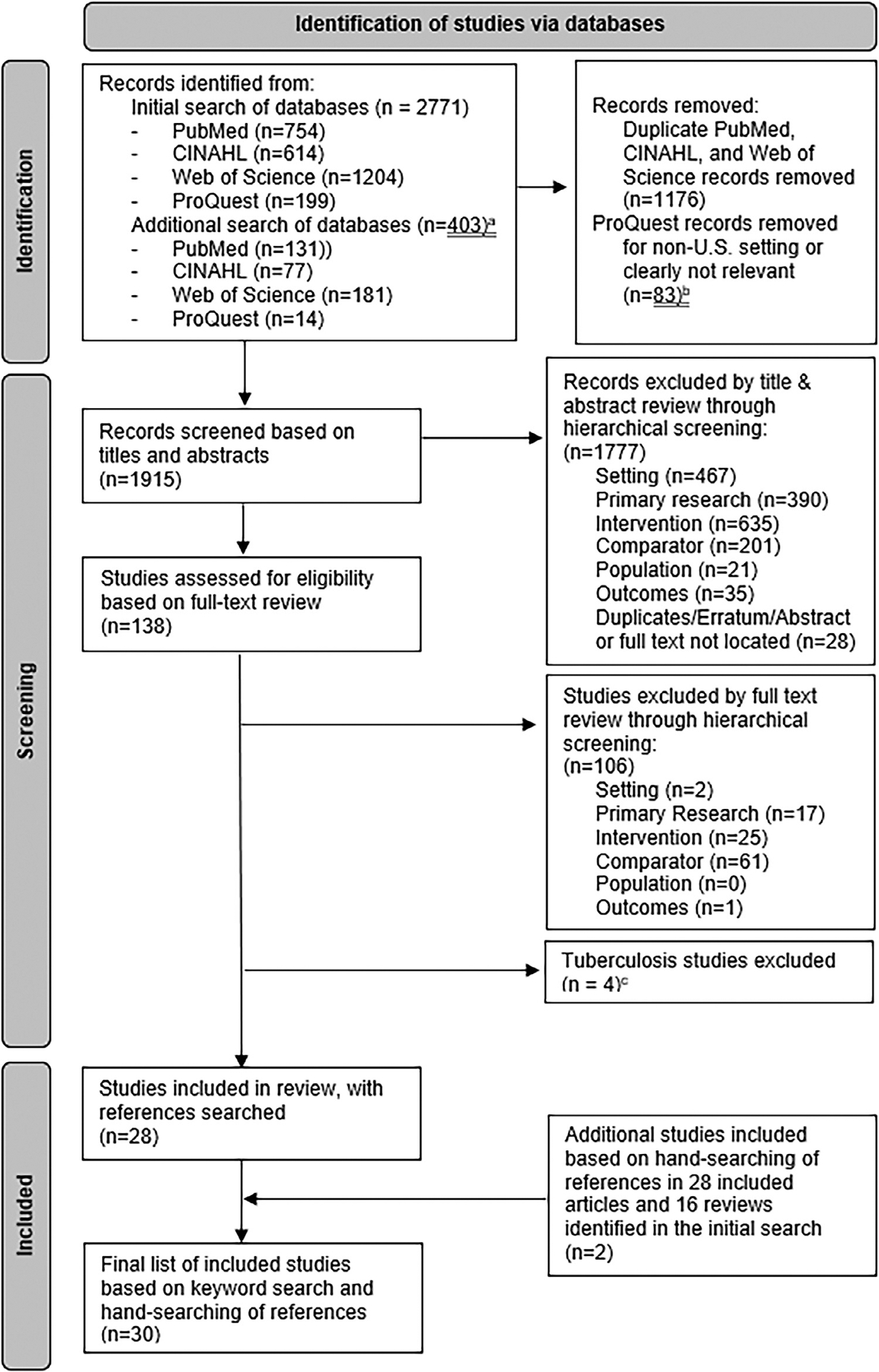
Flow diagram of included studies. ^a^After initial review, a second search was conducted with additional terms, as described in the methods. ^b^ProQuest records followed a slightly different screening process because many dissertations comprised 3 standalone studies. In an initial review of titles and abstracts, records that were clearly not US setting or relevant were excluded. Remaining records followed the title/abstract and full-text screening process using the hierarchical screening criteria. ^c^ As described in the methods, the original search strategy included studies of HIV, sexually transmitted infections (STIs), and tuberculosis. However, tuberculosis studies were subsequently excluded because the analyses of the effectiveness of disease intervention specialist (DIS) activities for tuberculosis were too dissimilar from that for HIV and STIs to allow for meaningful comparisons.

The keyword search yielded 1,915 unique records after removing duplicates, of which 1,777 were removed after screening titles and abstracts, 106 were removed after full-text review, and 4 were removed due to a focus on tuberculosis.^18–21^ The 28 included studies were supplemented with 2 studies based on hand searching references, yielding 30 studies (see Figure 1). A common study exclusion was an insufficient comparator. In the first stage (title and abstract review), among the 423 studies that met the first 3 hierarchical criteria of primary research, setting, and intervention, 201 (47.5%) were removed due to an unsuitable comparator. Among the 94 studies assessed in the full-text review that met the first 3 hierarchical criteria, 61 (64.9%) were removed due to an unsuitable comparator.

Table 1 summarizes study characteristics. Eleven studies focused on HIV exclusively,^23–33^ 15 focused on non-viral STIs exclusively,^34–48^ and 4 included both HIV and bacterial STIs.^49–52^ These studies examined contact tracing for HIV and syphilis,^49^ increasing the intensity of partner notification and cluster investigation for syphilis,^35,37^ partner notification or partner services,^23–25,27–29,36,40,48^ DIS-delivered expedited partner therapy for non-viral STIs,^38,44^ field-delivered therapy for gonorrhea and chlamydia,^45^ on-site placement of DIS for HIV^31^ or STIs,^46,47^ and modifications to the delivery of HIV/STI partner services such as telehealth or different interview techniques.^26,30,32–34,39,41–43,50–52^ The most common study design was an external control group,^23–26,29,33,36,40,42,49,50,52^ followed by pre-post designs without external control groups,^30,35,37,45–47,51^ RCTs,^28,34,38,41,44^ pre-post designs with external control groups,^27,31,32,39^ and external control group via pre-post designs.^43,48^ Studies were distributed regionally across the West,^24,26,27,34,38,41,42,45,46,48,50,51^ Northeast,^23,25,29–33,36,39,47^ and South;^28,35,37,43,44,49,52^ only 1 study was in the Midwest.^40^ Most studies were in urban locations, with the exception of 2 statewide studies^49,51^ and 3 studies assessing outcomes in multiple local health departments or counties.^28,36,38^ Two studies were linked, providing an analysis of the same intervention (intensified partner notification and cluster investigation for syphilis) using an identical study design and data source.^35,37^

Health outcomes among index patients were considerably more favorable with HIV/STI partner services than without. Persons with HIV reached by DIS had higher linkage to care and established care,^23^ HIV viral suppression,^49^ and condom use with notified partners.^27^ Compared to a reminder system, field follow-up yielded higher return rates for chlamydia treatment.^40^ Expanding STI partner services to all men who have sex with men (MSM) with bacterial STIs and testing their sex partners for HIV prior to case closure yielded increased HIV testing and new HIV diagnoses.^51^

Partner outcomes were also more favorable under HIV/STI partner services. Patients receiving HIV partner services were more likely to be linked to HIV care^26^ and notify partners,^24^ DIS elicited more partners per HIV index patient compared to community clinicians,^29^ and HIV partner notification was higher when completed by public health counselors versus by patients.^28^ Incorporating field testing into HIV partner services resulted in more notified partners testing for HIV.^30^ One state health department’s campaign to reduce syphilis yielded more persons treated prophylactically per index patient.^35,37^ One study had mixed results: among men with nongonococcal urethritis who received nurse referral, field follow-up, or DIS interviews without field follow-up, nurse referral counseling elicited the highest number of partners per index patient but field follow-up yielded more treated female sex partners per index patient.^40^

Studies examining population-level epidemiological outcomes found reductions in reported rates of gonorrhea. There were fewer repeat gonorrhea cases and incident diagnoses among civilians when gonorrhea patients treated at a military base received interviews and contact tracing under the direction of a health department representative,^48^ counties with a higher percent of partners brought to preventive treatment had lower reported gonorrhea rates,^36^ and reported gonorrhea rates were lower when health department staff reprioritized patients to prioritize high-morbidity geographical areas.^39^

There was mixed evidence for DIS-delivered expedited partner therapy and field-delivered therapy. There was an increase in chlamydia and gonorrhea index patients treated^45^ but no differences in their reported condom usage.^44^ The 2 studies examining partner outcomes had divergent findings: one reported increased patient-delivered therapy and decreased chlamydia test positivity and gonorrhea diagnoses,^38^ whereas the other reported no difference in time between female index patients’ initial visits until treatment of their male partners, no reduction in repeat trichomoniasis rates, and a lower percentage of male partners with verified treatment in the DIS group compared to the patient-delivered partner therapy group.^44^ The divergent findings are potentially attributable to differences in the strength of evidence (with improvements reported by the study with more suitable evidence for causal inference^44^) or different infections.

Most partner services outcomes improved after placing DIS in HIV clinics,^46^ an STI clinic,^47^ and healthcare facilities.^31^ However, the specific benefits were mixed. Although index patients were more likely to be interviewed^46,47^ and diagnosed earlier,^46^ there were no observed differences in index patient treatment.^46^ Two studies found mostly favorable outcomes among partners (such as partners elicited, notified, and treated)^31,46^ while another study reported no differences in partner-related outcomes.^47^ These divergent outcomes are not readily attributable to differences in suitability of causal evidence, infection, geography, or time period.

Two studies examined alternative interview techniques, finding that adding cues to aid recall yielded more partners elicited and located^34^ but that social network analysis interviews did not yield different partner elicitation or other partner outreach outcomes compared to traditional partner notification interviews.^43^ Studies comparing different delivery modes found that telephone partner services for HIV and syphilis had worse partner outcomes along the continuum compared to inperson partner services.^32,50^ Evidence on internet and text message partner services was mixed. One study compared both modes to standard HIV partner service, finding that success along the partner services continuum varied by notification type; the likelihood of contacting partners was highest for text messaging partner services, notifying contacted partners was highest for internet partner services, and testing notified partners for HIV was highest for traditional partner services.^33^ In an RCT, MSM receiving web-based partner notification services had fewer partners tested, although there was low power to detect differences due to the small sample.^41^ MSM enrolled in text message reminder services did not have different subsequent asymptomatic STI diagnosis rates compared to MSM without reminders.^42^ Compared to traditional HIV/STI partner services, email-based partner notification for pseudonymous sex partners for which email was the only available contact information resulted in fewer sex partners notified and tested.^52^ However, it is difficult to conclude definitely that text message reminders or email-based partner notification are ineffective because the text message reminder groups opted into the intervention and many “control” participants already used reminders,^42^ and the study of pseudonymous sex partners had a low sample size and did not adjust for differences between groups.^52^

Overall, the strength of the body of evidence on program effectiveness was low with respect to the study designs’ suitability for assessing the causal impact of partner services and other DIS-delivered interventions on clinical and epidemiological outcomes. One study had a strength of evidence rating of “high”; most studies were rated as “low” (n=18, 60%) or “medium” (n=11, 36.7%). (See Table 2 for the strength of evidence ratings and the Appendix for the JBI critical appraisal checklists.)

In addition to specific issues affecting the strength of evidence described in the preceding narrative description of study findings, there were few RCTs, which provide the highest level of causal evidence. Specific weaknesses in the RCTs were suspected errors in the reported results,^34,44^ a low sample size that led to a study’s early termination,^41^ and insufficient information for a complete assessment.^28^ Many studies using external control groups had non-equivalence of treatment groups and potential selection bias, such as comparing outcomes among those who accepted the intervention to those who did not, without appropriate statistical adjustment for individual-level characteristics or other confounders. Most studies with external control groups only examined “post” data, rather than collecting before-and-after data in both groups for a differences-in-differences design. In many instances, there was missing or unclear information about the study designs such as descriptions of the interventions delivered, potential spillover effects, participant attrition, selection biases, or sufficient details about the statistical analyses performed to allow for a comprehensive understanding of the approach and replication.

## DISCUSSION

Overall, this systematic review identified evidence across multiple studies and settings for the favorable impact of HIV/STI partner services on the continuum of index patient and partner outcomes, and on reported gonorrhea incidence. DIS-delivered expedited partner therapy and field-delivered therapy improved treatment of STI index patients but there was mixed evidence for partner-related outcomes. Most partner services outcomes improved after placing DIS in clinics. Compared to in-person delivery, telephone partner services yielded worse partner outcomes; however, there is insufficient evidence regarding internet-based or text message partner services.

The first evidence gap is the suitability of the evidence for assessing the causal impact of DIS-delivered interventions. Although there is a large literature on these interventions, many studies were excluded from this review because their designs were not suitable for impact evaluation, such as descriptions of program participants and program outcomes,^53–55^ comparisons of partner services outcomes between participants with different demographic characteristics,^56–58^ or not having a statistical test to assess the significance of comparison.^59–61^ Based on a holistic assessment of the study design, the critical appraisal checklists, and key limitations, almost all studies were rated as providing low or medium strength evidence. In the full-text review, approximately two-thirds of articles meeting the primary research, setting, and intervention criteria were excluded due to the lack of a suitable comparator for an impact evaluation. Many articles described the outcomes of specific outbreaks (e.g., number of index patients treated; number of contacts/partners identified, tested, and treated; and test positivity among contacts/partners) without a comparison group to assess how results would have differed in the absence of DIS-delivered interventions. Few studies used an RCT design; as noted by Kerani et al.,^41^ the difficulty of recruiting subjects limits the feasibility of implementing this study design. Another common limitation was not adjusting for group differences to ensure suitability for comparing treatment and control groups; for example, several studies provided unadjusted bivariate comparisons between groups despite nonequivalence of treatment arms. Similarly, studies with pre-post designs and external control groups did not employ methods that allow for strong causal inference.^27,31,32,39^ Another common methodological issue was the primary focus on program process measures such as testing and partners elicited; although these are important for understanding the indirect impact of partner services, only 5 studies examined outcomes such as future diagnoses and repeat infections.^36,38,39,44,48^

A second evidence gap is limited geographic representation. New York City and Seattle/King County were over-represented, corresponding to well-established investigators and study teams that frequently publish in this subject area. Notably, there was only one study from Texas^52^ and no studies from California or Florida, despite those states having a disproportionately high number of incident diagnoses of HIV and STIs.^62,63^ Evidence from additional locations, particularly from local health departments and rural settings that may have lower levels of staff and other resources, would allow for assessment of the extent to which findings are generalizable. It would improve the ability to assess whether the impact on clinical and epidemiological outcomes differs by setting.

As partner services programs evolve, there are several evidence gaps to prioritize. First, updating the scientific evidence with more recent data, research designs with higher internal validity, and improved adjustment for non-equivalent treatment groups and selection bias are important given changes in the epidemics that can influence outcomes such as reinfection and diagnosis rates and to support future modeling studies. Quantifying these outcomes is essential. Although RCTs can offer the highest level of evidence, they are often unrealistic in the context of limited funding for public health systems and services research^64^ and STI research,^65^ the length of time to execute studies, and pragmatic considerations. Alternative approaches include quasi-experimental designs such as synthetic matched controls or differences-in-differences using surveillance and programmatic data, or stepped-wedge designs whereby updated interventions are phased into different facilities or localities over time. Second, evidence on effectiveness could be supplemented with evidence on implementation strategies using research designs such as hybrid implementation-effectiveness trials. Delays in the uptake of health interventions are well-documented^66,67^; additional contextual challenges for HIV/STI prevention include chronic public health workforce shortages,^68^ critical gaps in public health infrastructure,^69^ and the backlash against public health,^70^ compounded by rising STI rates.^57^ As such, implementation science is a critical addition to impact evaluation to ensure that effective DIS-delivered interventions are integrated into practice within this broader context. Third, the literature would benefit from cross-disease approaches to assessing impact following the Centers for Disease Control and Prevention’s national strategic plan that emphasizes syndemic approaches to increase collaboration and service integration across infectious diseases.^71^ Although DIS provide services for multiple infections including tuberculosis and COVID-19, all included studies focused exclusively on HIV and/or STIs; the effectiveness of DIS intervention on other infections remain separate literatures. Only 4 studies included both HIV and STI outcomes; this is a missed opportunity given efforts to integrate services. A fourth area for future research is evaluating new partner services strategies, such as the extent to which molecular epidemiology for HIV/STI partner services improved clinical and population outcomes (versus the current literature which primarily uses the molecular data for descriptive epidemiological analyses), the role of new data systems following enhancements under the Data Modernization Initiative,^72^ new innovations developed during the COVID-19 pandemic such as enhanced use of telehealth services, and new strategies implemented as a result of the DIS Workforce Development Funding supplement.^73^

### Limitations

This systematic review had several limitations. First, this review did not include costs; some interventions that may be less effective, such as telephone-delivered partner services, may be more cost effective. Second, the assessment was limited to US settings due to variable terminology and program design across countries. Third, there is a possibility of missed studies due to inconsistent terminology across the infections and over time; however, the potential impact of this limitation is likely limited because the hand search yielded few new records and the study team included subject matter experts for each disease area. Fourth, it was infeasible to do a meta-analysis or formally assess the risk of publication bias due to the heterogeneity in study designs and reported outcomes. Fifth, DIS offer a range of services and benefits that are hard to measure; consequently, some outcomes may have been missed. Finally, for many studies, the information on study characteristics and key findings was unclear. This may have resulted in some studies being downgraded as lower quality evidence due to insufficient information on the study design. Similarly, it was infeasible to assess the rigor of the clinical and epidemiological outcomes due to incomplete information on how outcomes were measured. This is potentially because many studies were published before journals implemented study reporting guidelines.^74^ Given the long time horizon for included studies, it was infeasible to contact all study authors for clarification and missing information.

## CONCLUSIONS

DIS are a critical workforce for a reimagined public health system that can respond quickly to emerging infections.^75^ The findings on improved partner services outcomes after placing DIS in clinics and better outcomes with in-person versus telephone-based partner services suggests that although virtual program delivery can be beneficial particularly in rural areas, Ending the HIV Epidemic in the US jurisdictions, and local regions with provider deserts, technology may not always be an adequate substitute for in-person DIS involvement. As the role of DIS expands and partner services programs evolve to integrate services across disease siloes and incorporate new surveillance system technologies, program evaluations will be critical to demonstrate the value of these public health investments and identify areas for improvement. Opportunities to strengthen the evidence base include establishing a streamlined set of core metrics, implementing more rigorous causal inference methodologies to assess impact, linking data systems to integrate program and clinical outcomes data, and supplementing impact evaluations with evidence on implementation strategies.

## Supplementary Material

Appendix

## Figures and Tables

**Table 1.** Characteristics of studies examining the effectiveness of partner services for HIV and sexually transmitted infections

Study	Intervention and infection	Study design and comparison group	Data sources and time period	Study setting and population
Billock et al., 2021^49^	Intervention: DIS contact tracing services Infections: HIV, syphilis	Design: External control group Comparator: Previously diagnosed PWH who were not reached by DIS	Data: North Carolina Electronic Disease Surveillance system Time period: 01/2013–06/2017	Setting: North Carolina Population: MSM who were diagnosed with HIV or early syphilis Sample: *n*=2,350 (reached by DIS: *n*=1,397; not reached by DIS: *n*=953)
Bocour et al., 2013^23^	Intervention: Field Service Unit (FSU) HIV partner services Infection: HIV	Design: External control group Comparator: Non-FSU participating facilities	Data: NYC HIV Surveillance Registry and FSU database Time period: 2007–2011 (assumed; time period not clearly stated)	Setting: NYC (multiple facilities; details not specified) Population: Residents ≥13 years old newly diagnosed with HIV and reported to the NYC health department Sample: *n*=10,095 (FSU patients: *n*=4,108; non-FSU patients: *n*=5,987)
Brewer et al., 2005^34^	Intervention: Supplementing partner notification contact interviews with interviewing techniques that add recall cues: 1) cues with locations where people meet partners, role relationships, network ties, and first letters of names (“combined location/role/letter/network cue set”); and 2) cues including common first names (“first name cue set”) Infections: Chlamydia, gonorrhea, syphilis	Design: Randomized controlled trial Comparator: Standard cues referring to individual characteristics	Data: El Paso County health department STI program database Time period: 08/2000–06/2001	Setting: Colorado Springs, Colorado Population: Individuals diagnosed with bacterial STIs and who reported multiple sex partners in the past 90 days in interviews with county disease control staff Sample: *n*=123 (combined cues: *n*=35, first name cue set: *n*=41, individual characteristics cues [control]: *n*=47)
CDC, 1992^35^	Intervention: An intervention campaign by the state health department and CDC to reduce the incidence of early syphilis through increased partner notification and cluster investigation Infection: Syphilis	Design: Pre-post design with no external control group Comparator: 6 weeks before the campaign vs. the first 6 weeks of the campaign	Data: Alabama health department STI databases Time period: 6/17/1991–11/07/1991	Setting: Montgomery County, Alabama Population: Syphilis patients, their sex partners, and their associates at high risk Sample: *n*=373 patients, 984 sex partners, and 1,446 associates at high risk (sample sizes for the pre- versus post-period were not provided)
Du et al., 2007^36^	Intervention: Partner notification Infection: Gonorrhea	Design: External control group Comparator: Comparison of counties (which we classify in this review as “external control group”) with different levels of partner notification effectiveness (percent of patients interviewed, contact index, partner test positivity, percent of infected partners provided curative treatment, percent of partners unable to be located)	Data: Partner services and surveillance data Time period: 1992–2002	Setting: 15 urban counties in New York, excluding NYC Population: Diagnosed gonorrhea patients Sample: *n*=100,756 diagnosed index patients, *n*=37,393 interviewed patients, *n*=34,807 named sex partners; the sample size for each analysis differed depending on the exposure measure used
Engelgau et al., 1995^37^	Intervention: Intensified partner notification and cluster investigation campaign that increased the number of public health workers assigned to syphilis control activities and hours of STI clinical services; public health workers also received supplemental training and intense supervision on intensified partner notification and cluster investigation techniques Infection: Syphilis	Design: Pre-post design with no external control group Comparator: 6 weeks before the campaign versus the first 6 weeks of the campaign	Data: Montgomery County STI program records Time period: Campaign was implemented 06/17/1991–11/7/1991; study period was 6 weeks before the campaign versus the first 6 weeks of the campaign	Setting: Montgomery County, Alabama Population: Syphilis patients, their sex partners, and their associates in the social network at high risk Sample: Early intervention period (first 6 weeks), *n*=151 patients, 390 partners, 636 associates at high-risk; control period (6 wk pre-campaign period), *n*=78 patients, 178 partners, 183 associates at high risk
Golden et al., 200924	Intervention: Partner notification services delivered by public health staff Infection: HIV	Design: External control group Comparator: No partner services	Data: Patient surveys from behavioral surveillance program Time period: 2006–2007	Setting: 1 HIV clinic in Seattle, Washington Population: Random sample of English-speaking HIV patients Sample: *n*=370 (among eligible participants who attended their clinic appointment, 85% consented to study participation)
Golden et al., 2015^38^	Intervention: Public health expedited partner therapy, comprising local health jurisdictions’ promotion of patient-delivered partner therapy (PDPT) use and prioritized provision of public health partner services Infections: Chlamydia, gonorrhea	Design: Stepped-wedge, community-level randomized design Comparator: Comparison of outcomes between communities that had received the intervention versus those that had not yet received it and within communities (before and after the intervention)	Data: Laboratory data from Infertility Prevention Project and Planned Parenthood of Western Washington clinics; case report forms from medical providers and laboratories; partner services data; and interviews with randomly selected patients Time period: 10/2007–08/2009	Setting: 23 local health jurisdictions in Washington State Population: Heterosexual patients diagnosed with gonorrhea or chlamydia Sample: No sample size reported for the overall study or by treatment group because the study was a community-level randomized trial
Halkitis et al., 2011^25^	Intervention: Partner services Infection: HIV	Design: External control group Comparator: Alternative venue testing via mobile van and social network strategy through inter-agency referrals by 1 community-based organization (note: for our review, we considered these as comparators because they were delivered by investigators rather than DIS or other public health staff)	Data: NYC health department monthly reports of HIV testing data for African American MSM (partner services strategy); cross-sectional surveys and rapid HIV antibody tests administered by investigators (alternative venue testing and social network strategies) Time period: 04/2008–08/2009	Setting: NYC Population: African American MSM aged 18–64 y with previously undiagnosed HIV infection Sample: Partner services, *n*=49; alternative venue testing, *n*=400; social networks strategy, *n*=109
Han et al., 1999^39^	Intervention: Reprioritizing to focus on interviewing patients within high-morbidity geographical areas (“core-targeting interventions”) Note: During a syphilis epidemic, field staff were diverted from gonorrhea to syphilis control activities, described as “reduced-core.” Infection: Gonorrhea	Design: Pre-post design with external control group Comparator: Syracuse, New York (traditional field services)	Data: STI surveillance data and aggregated partner services interview data Time period: 1975–1997 (pre-intervention: 1975–1983, initial full-core: 1984–1988, reduced-core during the syphilis initiative: 1989–1991, full-core: 1992–1997)	Setting: Buffalo/Erie County, New York (intervention) and Syracuse/Onondaga County, New York (control) Population: Residents with reported gonorrhea Sample size not specified; this was a population-based study
Heumann et al., 2017^50^	Intervention: In-person partner services interviews Infections: HIV, syphilis	Design: External control group Comparator: Telephone partner services interviews	Data: Partner services records and the enhanced HIV/AIDS Reporting System (eHARS) Time period: 2010–2014	Setting: King County, Washington Population: All residents with early syphilis or newly diagnosed HIV infection who were interviewed by DIS for partner services Sample: early syphilis, *n*=1,328 (in-person interviews [treatment]: *n*=682; telephone interviews [control]: *n*=646); HIV, *n*=847 (in-person interviews [treatment]: *n*=358; phone interviews [control]: *n*=489)
Hood et al., 2017^26^	Intervention: Partner services, following the integration of HIV surveillance and field services Infection: HIV	Design: External control group Comparator: Newly diagnosed PWH who did not receive partner services	Data: HIV surveillance and field services data Time period: 2010–2015	Setting: King County, Washington Population: All PWH who entered the King County HIVsurveillance system from 2010–2015 Sample: *n*=1,474 (treatment: *n*=1,187; control: *n*=287)
Hoxworth et al., 2003^27^	Intervention: Partner notification Infection: HIV	Design: Pre-post design with external control group (although this is a pre-post design, the extracted results comprise “post” comparisons between groups) Comparator: Patients at 1 Denver HIV counseling and testing site who received a negative HIV test but were at high risk for HIV infection; they did not receive partner notification	Data: Study interviews at baseline, 3 months, and 6 months Time period: 09/1998–04/2000 for recruitment; full study period not clearly specified	Setting: Denver, Colorado Population: Index patients with HIV who were previously interviewed to identify partners, partners who the health department notified regarding HIV exposure, and patients at 1 Denver HIV counseling and testing site who received a negative HIV test but were at high risk for HIV infection (controls) Sample: *n*=202 (70 index patients, 33 notified partners, and 99 controls)
Katz et al., 2016^51^	Intervention: Revising health departments’ STI partner services programs to provide services to all MSM with early syphilis, gonorrhea, or chlamydia and test all MSM and their sex partners for HIV prior to index patient case closure Infections: HIV, syphilis, gonorrhea, chlamydia	Design: Pre-post design with no external control group Comparator: Pre-intervention period	Data: Matched HIV and STI surveillance and partner services data Time period: 01/2010–10/2014	Setting: Washington State Population: MSM with early syphilis, gonorrhea, or chlamydia without a prior HIV diagnosis Sample: *n*=8,133 (pre-intervention: *n*=3,253; intervention period: *n*=4,880)
Katz et al., 1988^40^	Intervention: Study 1: Field follow-up for patients with chlamydial infection; Study 2: (a) DIS interviews with index patients to elicit partners and encourage patients to refer partners to the clinic and (b) DIS interviews with index patients and field follow-up to named partners Infection: Chlamydia	Design: External control group; although the external groups differed for the 2 studies, the same design was used Comparator: Study 1: Reminder system to come to clinic for chlamydia treatment; Study 2: Nurse counseling of treated patients to encourage them to refer partners for treatment	Data: Partner services program data from an STI clinic Time period: 08/1985–12/1985	Setting: Indianapolis, Indiana Population: Study 1: Men and women at a county STI clinic with diagnosed chlamydial infection; Study 2: female sex partners of male STI clinic patients with nongonococcal urethritis Study 1: N=142 (76 women, 66 men); Study 2: N=678 male index patients
Kerani et al., 2011^41^	Intervention: Patient-delivered partner therapy (PDPT) and a web-based partner notification service (inSPOT) with 3 treatments (PDPT, inSPOT, and combined PDPT/inSPOT) Infections: Gonorrhea, chlamydia	Design: Randomized controlled trial Comparator: Standard partner management	Data: Partner services program data; baseline and follow-up study interviews approximately 2 wk apart Time period: 07/2007–03/2009 (note: the trial ended early due to low enrollment)	Setting: King County, Washington Population: MSM with gonorrhea and chlamydia eligible for partner services, excluding MSM diagnosed with HIV or syphilis Sample: PDPT only, *n*=13; inSPOT only, *n*=10; PDPT/inSPOT combined, *n*=17; standard partner management (control), *n*=13 (among eligible MSM, 75 [19%] consented to the study and 53 [71%] of enrolled MSM completed the baseline and follow-up interviews)
Landis et al., 1992^28^	Intervention: Partner notification by public health counselors Infection: HIV	Design: Randomized controlled trial Comparator: Partner notification by patients	Data: North Carolina HIV testing program data Time period: 11/1988–06/1990	Setting: 3 large county health departments in North Carolina Population: Clients with confirmed HIV infection identified through anonymous testing or confidential testing through the health departments, excluding individuals who previously tested positive for HIV or had no new sex or needle-sharing partners Sample: *n*=74 index patients and 310 partners (treatment group, *n*=39 index patients and 157 partners; control group: *n*=35 index patients and 153 partners); among those eligible for the study, 45.7% enrolled
Malave et al., 2008^29^	Intervention: Partner notification provided by DIS in STI clinics Infection: HIV	Design: External control group Comparator: Partner notification delivered by community clinicians in non-STI clinics	Data: Provider report form from community clinicians and the NYC health department HIV surveillance registry Time period: 2004	Setting: 10 health department-run STI clinics and other diagnosing facilities (hospitals, clinics, and private physician offices) in NYC Population: Index patients diagnosed with HIV, excluding those diagnosed in health department-run TB clinics and NYC jail clinics Sample: *n*=3,666 (diagnosed in STI clinics, *n*=206; diagnosed in non-STI clinics, *n*=3,460)
Renaud et al., 2011^30^	Intervention: Incorporating field testing into partner services Infection: HIV	Design: Pre-post design with no external control group Comparator: Pre-intervention period	Data: Partner services data, provider reports, partner-provided test results documents, medical records Time period: 09/2006–08/2007 (pre-intervention) versus 09/2008–08/2009 (intervention)	Setting: NYC Population: Sex and needle sharing partners of recently diagnosed HIV patients Sample: *n*=532 partners notified (pre-intervention period, *n*=181; intervention period, *n*=351)
Ronen et al., 2019^42^	Interventions: Quarterly text message service for testing reminders to MSM with early syphilis and gonorrhea or chlamydia (note: the study includes a third group of MSM already enrolled in another reminder service, which we interpreted as a second treatment group because they were compared to the no-text message group) Infections: Chlamydia, gonorrhea, syphilis	Design: External control group (note: there were pre-intervention data but the comparison between groups is based on post-intervention data only) Comparator: MSM who did not accept the intervention and did not use text message reminders	Data: STI surveillance and partner service data Time period: 07/2013–01/2018	Setting: King County, Washington Population: Individuals identified as cis or trans male gender and who reported sex with men in the past year, had male sex partners recorded by their provider on the case report, and/or were diagnosed with rectal gonorrhea or rectal chlamydia Sample: *n*=3,376 (text message reminder accepted [treatment 1]: *n*=521; no text message reminder [comparator]: *n*=2,629; already enrolled in another reminder service [treatment 2]: *n*=226)
Rosenberg, 1997^43^	Intervention: Social network analysis (SNA) interviews conducted by specially trained interviewers Infection: Syphilis	Design: External control group via a pre-post design (different interventions were delivered in the pre and post periods) Comparator: Traditional partner notification interview	Data: Partner notification and SNA interview data Time period: Partner notification, 01/01/1996–06/30/1996; SNA: 08/01/1996–01/31/1997	Setting: 4 contiguous parishes in Louisiana: Ascension, Iberville, East Baton Rouge, and West Baton Rouge Population: Partner notification index patients were primary and secondary (P&S) syphilis patients from the state surveillance database residing within the 4 parishes; SNA index patients were a convenience sample of P&S syphilis patients that had not yet received contact tracing or partner notification interviews Sample: *n*=88 index patients (partner notification, *n*=72; SNA, *n*=16) and 80 contacts from 10 of the 16 SNA index patient interviews
Schwebke & Desmond, 2010^44^	Interventions: 1) Patient delivered partner therapy (PDPT) and 2) DIS interview and DIS-assisted partner notification and treatment Infection: Trichomoniasis	Design: Randomized controlled trial Comparator: Patient self-referral of partners (partner referral)	Data: Repeat laboratory specimens collected at the clinic and study questionnaires Time period: 02/2003–06/2008	Setting: Jefferson County health department STI clinic, Birmingham, Alabama Population: Adult women with *Trichomonas vaginalis* infection (primarystudy population), and their male partners Sample: *n*=484 women (PDPT, *n*=162; DIS, *n*=162; partner referral [control], *n*=160) and 115 male partners (70 enrolled from the DIS arm, 45 enrolled from the partner referral arm; information from the 143 males receiving PDPT medication were via self-report by the female index patients) (continued on next page)
Steiner et al., 2003^45^	Intervention: Field-delivered therapy Infections: Chlamydia, gonorrhea	Design: Pre-post design with no external control group Comparator: Pre-intervention period	Data: San Francisco health department STI program Time period: 1998–2001 (note: the statistical test considered in our review is for the 1998 to 2000 comparison)	Setting: San Francisco, California Population: Persons diagnosed with uncomplicated chlamydial infection and/or gonorrhea who were unable or unlikely to come into the municipal STI clinic for treatment Sample: *n*=1,062 (pre-intervention: *n*=432 [1998]; post-intervention: *n*=630 [2000])
Taylor et al., 2010^46^	Intervention: Placement of on-site or on-call DIS in 3 HIV clinics to deliver penicillin to patients and partners and conduct on-site partner elicitation interviews Infection: Syphilis	Design: Pre-post design with no external control group Comparator: Pre-intervention period	Data: STI surveillance data Time period: 01/2006–01/2008 (pre-intervention) versus 02/2008–09/2009 (post-intervention)	Setting: 3 HIV clinics in Maricopa County, Arizona Population: Patients diagnosed with syphilis Sample: *n*=334 (before DIS placement: *n*=219; after DIS placement: *n*=115)
Tributino et al., 2018^47^	Intervention: Provision of on-site DIS-delivered partner notification services as part of standard clinic care at an STI clinic Infections: Gonorrhea, syphilis	Design: Pre-post design with no external control group Comparator: Pre-intervention period	Data: Medical records from the STI clinic Time period: 08/2014–12/2015, excluding the implementation month (04/2015)	Setting: 1 public health STI clinic, Providence, Rhode Island Population: Patients who received a diagnosis of gonorrhea or syphilis Sample: *n*=145 (pre-intervention: *n*=58; post-intervention: *n*=87)
Udeagu et al., 2012^31^	Intervention: Partner services delivered in HIV clinics by placing DIS on-site in hospitals and community providers (Field Services Unit [FSU]-participating sites) Infection: HIV	Design: Pre-post design with external control group (note: authors compared pre-post outcome separately for the treatment and control groups) Comparator: Partner services delivered by community providers that did not have the collaboration with on-site DIS	Data: Partner service data (HIV FSU database; Provider Report Form database) Time period: 2005–2008	Setting: Multiple facilities in NYC (details not specified) Population: Newly diagnosed HIV patients Sample: *n*=2,695 (FSU participating sites: *n*=678 pre-intervention, *n*=602 post-intervention; non-participating sites: *n*=788 pre-intervention, *n*=635 post-intervention)
Udeagu et al., 2014^32^	Intervention: Telephone partner services by the Field Services Unit Infection: HIV	Design: Pre-post design with external control group (note: the statistical analysis compared “post” outcomes between groups) Comparator: In-person partner services	Data: NYC Field Services Unit data Time period: 2009–2012	Setting: NYC Population: Sex partners named by HIV patients who had an interview with DIS and were HIV negative or with unknown HIV serostatus at notification Sample: *n*=3,604 (in-person notification: *n*=2,086; telephone notification: *n*=1,518)
Udeagu et al., 2014^33^	Interventions: Internet-based partner services and text messaging partner services (2 interventions) Infection: HIV	Design: External control group Comparator: Traditional partner services using postal mail, telephone calls, and field visits	Data: NYC Field Services Unit partner services data Time period: 01/2011–10/2012	Setting: NYC Population: HIV-diagnosed patients and their partners named during partner services investigations Sample: *n*=3,319 partners named by 1,828 patients (traditional partner services [control]: 2,604 partners named by 1,596 index patients; internet-based partner services [treatment]: 275 partners named by 73 index patients; text message-based partner services: 368 partners named by 176 index patients)
Vest et al., 2007^52^	Intervention: Provider partner notification via email for pseudonymous sex partners with email as the only contact information available Infections: HIV, syphilis	Design: External control group Comparator: Conventional method of partner notification, which uses contact information other than email addresses	Data: Austin/Travis County health department STI surveillance data Time period: 01/2004–06/2006	Setting: City of Austin and Travis County, Texas Population: Residents diagnosed with HIV and/or early syphilis who reported having sexual contact with pseudonymous email partners Sample: *n*=318 (treatment: *n*=53; control: *n*=265)
Woodhouse et al., 1985^48^	Intervention: Interviewing diagnosed patients and contact tracing, with the military clinics under direction of a health department representative Infection: Gonorrhea	Design: External control group via a pre-post design (different interventions were delivered in the pre and post periods); results stratified by military versus civilian populations Comparator: Rotating medical personnel without specialized STI training providing patient interviewing or counseling without partner elicitation or field follow-up	Data: Patient interview records and STI surveillance data (note: data sources presumed and difficult to interpret due to older study year) Time period: 1977–1979 (pre-intervention) and 1980–1982 (intervention)	Setting: Colorado Springs, Colorado (military base and surrounding community) Population: Military and civilian gonorrhea patients and their contacts Sample: Military population, *n*=3,842 gonorrhea patients (pre: *n*=1,934, post: *n*=1,908) and *n*=4,450 contacts (pre: 1,341, post: 3,109); civilian population, *n*=5,253 gonorrhea patients (pre: *n*=2,919, post: *n*=2,334) and *n*=7,502 contacts (pre: 3,212, post: 4,290)

CDC, Centers for Disease Control and Prevention; DIS, disease intervention specialists; FSU, Field Service Unit; HIV, human immunodeficiency virus; ILOW, indigenous leadership outreach workers; MSM, men who have sex with men; NYC, New York City; PDPT, patient-delivered partner therapy; PWH, persons with HIV; STI, sexually transmitted infection; SNA, social network analysis; TB, tuberculosis.

**Table 2.** Summary of studies examining the effectiveness of partner services for HIV and sexually transmitted infections

Study	Findings on key outcomes	Strength of Evidence for Impact Evaluation
Billock et al.^49^	The 6-month cumulative incidence of HIV viral suppression was 13.1 percentage points higher among previously diagnosed PWH reached by DIS versus PWH not reached by DIS (95% CI: 8.8–17.4). The adjusted cumulative incidence difference in 12-month HIV viral suppression was 6.7 percentage points higher among PWH reached by DIS versus PWH not reached by DIS (95% CI: 2.1–11.3).	Low
Bocour et al.^23^	79% of Field Services Unit (FSU) patients were linked to care within 91 days of HIV diagnosis versus 66% of non-FSU patients (*p*<0.0001); adjusted prevalence ratio=1.10 (95% CI: 1.08–1.12). 87% of FSU patients had established HIV care versus 84% of non-FSU patients (*p*=0.0001); adjusted prevalence ratio=1.04 (95% CI: 1.02–1.06).	Low
Brewer et al.^34^	A higher number of partners per STI patient were elicited after use of cues with the combined location/role/letter/network cues (mean=0.57) versus first-name cues (mean=0.29) and individual characteristics (mean=0.28) cues. The combined cues and first-name cues yielded on average 0.11 and 0.10 additional located partners per STI patient, respectively, compared to individual cue characteristics (mean=0 partners located). Note: For both findings, the statistical significance is implied but not clearly reported.	Medium
CDC^35^	The average number of infected persons per syphilis patient was not significantly different in the early syphilis campaign versus precampaign periods. The average number of persons prophylactically treated per syphilis patient was higher in the early syphilis campaign versus precampaign period (3.9 vs. 2.5, *p*<0.01).	Low
Du et al.^36^	The association between the percent of patients interviewed, contact index, and percent of partners not located (exposures) and the gonorrhea incidence rate (outcome) were not statistically significant in cross-sectional or longitudinal analysis. (Note: Although the authors highlighted a significant and positive association between the percent of partners unable to be located in the prior year and the gonorrhea incidence rate, this association only held for one of the 3 cross-sectional analyses and not the longitudinal analysis.) Counties with a higher percent of partners brought to preventive treatment (exposure) had a lower gonorrhea incidence rate in longitudinal analysis (rate ratio=0.94, *p*<0.0001) but this association was not statistically significant in cross-sectional analyses. Counties with a higher proportion of infected partners brought to treatment had a higher gonorrhea incidence rate in longitudinal analysis (rate ratio=1.10, *p*<0.01); this association was also statistically significant in cross-sectional analyses.	Medium
Engelgau et al.^37^	The average number of infected persons identified per index patient was not statistically significant between the early syphilis campaign period versus the pre-period. The average number of persons prophylactically treated per index patient was higher in the early syphilis campaign period versus the pre-period (3.9 vs. 2.5; *p*<0.01).	Low
Golden et al.^24^	Patients who received HIV partner services were more likely to notify ≥1 partner (68% vs 45%; adjusted odds ratio=3.2; 95% CI: 2.0, 5.3). Patients who received HIV partner services notified more partners (received partner services, mean=1.6, median=1; did not receive partner services, mean=1.1, median=0; *p*=0.0004).	Low
Golden etal.^38^	The percentage of patients receiving patient-delivered partner therapy from their diagnosing provider increased from 18.3% to 34.0% (*p*<0.001) The chlamydia test positivity among women aged 14–25 years that were tested in Infertility Prevention Project clinics within participating local health jurisdictions decreased from 8.2% to 6.5% (*p*<0.001). The annual rate of gonorrhea diagnoses among women aged 14–25 years declined from 59.6 to 26.4 per 100,000 (p<0.001). There was a non-significant 10% decline in chlamydia positivity and gonorrhea incidence among women aged 14–25 years after adjusting for temporal trends.	High
Halkitis et al.^25^	There were differences in the HIV test positivity rates for MSM by treatment arm (alternative venue testing: 6.3%, social networks strategy: 19.3%, partner services: 14.3%; *p*<0.001). In between-group comparisons, there was no statistically significant difference in HIV test positivity between the social networks strategy compared to partner services, a lower HIV test positivity between alternative venue testing compared to partner services (OR=0.40, 95% CI= 0.16, 0.98), and a lower HIV test positivity between alternative venue testing compared to social networks strategy (OR= 0.28, 95% CI= 0.15, 0.52).	Low
Han et al.^39^	The authors provide evidence that the gonorrhea incidence rate was lower during the “initial full-core” and “full core” periods (prioritizing high-morbidity geographical areas, excluding the period when field staff were diverted to syphilis) compared to both the pre-intervention period and the external control county. However, the statistical models were not clearly specified, making it difficult to summarize effect sizes.	Medium
Heumann et al.^50^	Early syphilis: Compared to telephone interview participants, in-person interview participants had more partners named (contact index, aRR: 1.68; 95% CI: 1.55,1.82; *p*<0.001), notified (notification index, aRR: 1.39; 95% CI: 1.24,1.56; *p*<0.001), tested for syphilis (syphilis test index, aRR: 1.34; 95% CI: 1.16,1.54; *p*<0.001), tested for HIV (HIV test index, aRR: 1.45, 95% CI: 1.14,1.86; *p*=0.003), and treated for syphilis (epidemiologic index, aRR: 1.19; 95% CI: 1.03,1.37; *p*=0.017). There were no significant between-group differences in the number of partners diagnosed with syphilis or HIV (syphilis case-finding indexand HIV case-finding index) or partners diagnosed with and treated for syphilis (brought-to-treatment index). HIV: Compared to telephone interview participants, in-person interview participants had more partners named (contact index, aRR=1.38; 95% CI= 1.18, 1.62; *p*<0.001), notified (notification index, aRR=1.24, 95% CI= 1.03, 1.50; *p*=0.026), and diagnosed with HIV (HIV case-finding index, aRR=2.17; 95% CI= 1.04, 4.50; *p*=0.039). There were no significant between-group differences in the number of partners tested for HIV (HIV test index).	Medium
Hood et al.^26^	PWH who received partner services had higher linkage to HIV care within 30 and 90 days of diagnosis, compared to PWH who did not receive partner services (30 days, aRR=1.10, *p*=0.004; 90 days, aRR=1.07, *p*=0.014).	Low
Hoxworth et al.^27^	During the follow-up period, there was a higher proportion of using condoms during each vaginal or anal sex episode among index patients with HIV infection with notified partners (80%) and notified partners with index patients (100%) compared to index patients with non-notified partners (50%), notified partners with other persons (38%), and controls (30%) (*p*<0.05). A higher proportion of vaginal or anal sex episodes were protected for index patients with notified partners (85%) and notified partners reporting partnerships with index patients (100%) than in other groups (55%, 37%, and 41%, respectively; *p*=0.002). During the follow-up period, there were no statistically significant differences in the numbers of sex partners or new sex partners between groups.	Low
Katz et al.^51^	HIV testing among MSM with bacterial STIs increased from 63% to 91% (*p*<0.001); this was statistically significant after adjusting for provider type, county, and STI type(*p*<0.001). After adjusting for provider type, county, STI type, and temporal trends in HIV incidence, there was a marginally significant increase in the proportion of MSM with bacterial STIs newly diagnosed with HIV infection (aRR=1.34, *p*=0.07). The proportion of new HIV diagnoses among MSM concurrently diagnosed with a bacterial STI increased from 6.6% to 13% (RR=1.99, *p*<0.0001). Among MSW with gonorrheal infection who received partner services, HIV testing at the time of STI diagnosis or treatment increased from 27% to 71% (*p*<0.0001).	Low
Katz et al.^40^	Study 1: There was a higher rate of return for follow-up treatment among male and female patients with chlamydia with field follow-up (97%) versus a reminder system (79%) (*p*<0.001). Study 2: Among men with nongonococcal urethritis, nurse referral counseling to encourage partner notification yielded the highest number of partners elicited per index patient (1.16), followed by field follow-up (0.80, *p*=0.007) and DIS interviews without field follow-up (0.75, *p*=0.003). Study 2: Among men with nongonococcal urethritis, field follow-up yielded more treated female sex partners per index patient (0.72) compared to nurse referral counseling (0.22, *p*<0.001) or DIS interviews without field follow-up (0.18, *p*<0.001).	Medium
Kerani et al.^41^	There were no statistically significant differences by treatment arm in the mean number of partners elicited per STI index patient. There were no statistically significant differences by treatment arm in the mean number of partners notified per STI index patient. Participants receiving PDPT had higher mean number of partners treated compared to those not receiving PDPT (ratio of unadjusted means, 1.53, *p*<0.05), with no difference between those receiving web-based partner notification service (inSPOT) versus no inSPOT receipt. Participants receiving inSPOT had fewer partners tested for HIV versus participants with no inSPOT receipt (ratio of unadjusted means, 0.42, *p*<0.05), with no difference between PDPT versus no PDPT. There were no differences in the mean number of partners tested for syphilis by treatment arm. Patients receiving PDPT had a higher mean number of partners treated after adjusting for inSPOT assignment (ratio of adjusted means=1.54, *p*<0.05).	Low
Landis et al.^28^	A higher percent of elicited partners was notified in the treatment group (HIV partner notification by public health counselors) compared to the control group (HIV partner notification by patients) (50% versus 6.5%, *p*<0.001). Among the partners located, there were no statistically significant differences in the percent of partners tested between treatment and control groups. Among the partners located and tested, there were no statistically significant differences in the percent of partners that tested positive for HIV.	Medium
Malave et al., 2008^29^	DIS elicited more partners per HIV index case-patients than community clinicians (elicited ≥1 partner: 51.0% vs 17.7%, *p*<0.01; partner index: 0.87 vs 0.22, *p*<0.01). The proportion of new HIV diagnoses was similar between partners elicited by DIS and community clinicians.	Medium
Renaud et al.^30^	A higher proportion of notified partners tested for HIV during the intervention period (52% versus 76%, *p*<0.001); this finding was consistent across age groups, sex, and race/ethnicity. Although there were more HIV tests, the HIV seroprevalence of tested partners was not statistically different during the intervention period.	Low
Ronen et al.^42^	Relative to MSM who did not enroll to receive an text message reminder, MSM who received a text message reminder or who were already enrolled in another reminder service did not have a significant difference in subsequent asymptomatic STI diagnosis within 1 to 12 months after adjusting for demographics, HIV/PrEP status, STI, health insurance status, diagnosing provider, and calendar year (text message reminder group, aRR=0.80, *p*=0.13; already enrolled in another reminder service group, aRR=1.08, *p*=0.68)	Low
Rosenberg^43^	The social network analysis and traditional syphilis partner notification interviews yielded no significant differences in partner elicitation (number of named sex contacts, number of contacts with insufficient information to initiate follow-up activities, or number of contacts that were unable to be located). The 2 interview types yielded no significant difference in outcomes of partner outreach (number of newly infected contacts, number of contacts that refused the interview, or number of contacts previously treated).	Low
Schwebke & Desmond^44^	Repeat trichomoniasis infection rates among women were not statistically different between the either treatment group (DIS or PDPT) versus to the patient referral (control) group during the 1-month and 3-month follow-up visits. However, the DIS group had higher repeat trichomoniasis infection rates compared to the PDPT group (RR=1.54, *p*=0.03) at the 1-month follow-up visit; this between-group difference was not statistically different at the 3-month follow-up visit. (Note: This calculation is internally inconsistent; our calculation is that the 1-month reinfection relative risk comparing the DIS and PDPT groups was 2.60.) The reported condom usage rates among women were not statistically different between the treatment and control groups at the 1-month and 3-month follow-up visits. There was no statistically significant difference in means for the time elapsed from the female index patient’s initial visit to the known treatment date of their male partners. The percentage of female index patients’ male partners with verified treatment was higher in the PDPT group than the DIS or patient referral group (*p*<0.001).	Medium
Steiner et al.^45^	Between 1998 and 2000, there was a 31.0% relative increase in chlamydia and gonorrhea patients treated (*p*<0.001). Between 1998 and 2000, there was a statistically significant relative increase in STI patients treated except for persons aged 30–34 (relative increase=18.1%, *p*=0.090), Asian/Pacific Islander persons (relative increase: 20.1%, *p*=0.052), and MSM (relative increase: 2.1%, *p*=0.631). (Note: Although there were meaningful relative increases for persons aged 30–34 years and Asian/Pacific Islander persons, these demographic groups had the smallest sample size. Among MSM, the treatment completion was very high in 1998 (92.9%) so although this improved slightly to 94.8%, there was limited room for improvement.)	Low
Taylor et al.^46^	After DIS placement, syphilis patients were less likely to be diagnosed as late latent stage (26% versus 14%, *p*=0.02). After DIS placement, syphilis patients were more likely to be interviewed (94% versus 81%, *p*=0.001). After DIS placement, more partners per interviewed patient were initiated for investigation (1.1 versus 0.6, *p*=0.004) although there was no significant pre-post difference in the number of partners reported per patient interview. After DIS placement, there was a reduction in the number of days between diagnosis and interview among index patients (9 versus 18, *p*=0.02). After DIS placement, there was an increase in the number of partners initiated for investigation (0.6 versus 1.1, *p*=0.004). After DIS placement, there was an increase in the number of partners per index patient treated for syphilis exposure and/or infection (0.3 versus 0.6, *p*=0.02). After DIS placement, the mean time to syphilis treatment among partners decreased (21 days versus 8 days, *p*=0.007). There were no significant pre-post differences in the percent of index patients treated, the time to treatment of index patients, or the number of partners reported.	Medium
Tributino et al.^47^	The percentage of interviewed gonorrhea and syphilis index patients increased from 76% to 92% (*p*=0.007). The proportion of interviewed STI patients that were interviewed on the day of diagnosis increased from 61% to 85% (*p*=0.003). There was no significant difference in the average number of partners named. There was no significant difference in the percentage of STI index patients with at least one partner treated or the average number of treated partners.	Low
Udeagu et al.^31^	In participating sites, the HIV partner index ratio (partners elicited/patients interviewed) improved significantly after the FSU intervention (0.3 in 2005 versus 0.9 in 2008; *p*<0.0001). Newly diagnosed persons with HIV were more likely to have a submitted provider report form in participating sites than nonparticipating sites (75% versus 44%; *p*=0.0001) More partners were elicited in participating sites than nonparticipating sites (474 versus 13; *p*=0.0001). More partners were notified in participating sites than nonparticipating sites (194 versus 1; *p*=0.0001).	Low
Udeagu et al.^32^	Compared to in-person-notified partners of HIV patients, telephone-notified partners were less likely to test for HIV during or shortly following notification (40% vs 81%, *p*<0.0001).	Low
Udeagu et al.^33^	The partner contact rate was higher for text message HIV partner services (77%) compared to traditional HIV partner services (69%) and internet HIV partner services (41%; *p*<0.0001). The likelihood of notifying contacted partners was higher for internet HIV partner services (OR=2.1) and text message HIV partner services (OR=2.4) compared to traditional HIV partner services (*p*≤0.0001). The notification rate did not differ statistically between text message partner services versus internet partner services. The proportion of partners that tested for HIV after notification was higher for traditional partner services (69%) versus internet partner services (34%) or text message partner services (45%) (*p*≤0.0001). The proportion of notified partners that accepted HIV testing did not differ statistically between the text message and internet partner services groups. Note on interpretation of results: internet partner services were for persons who only provided an email address or other internet-based contact information, text message partner services were for persons who only provide a phone number, and traditional partner services included postal mail, telephone calls, field visits, and other contact information from the internet.	Medium
Vest et al.^52^	HIV and syphilis index patients’ pseudonymous email sex partners were less likely to be notified (49.7% versus 69.7%, *p*<0.001). Among notified partners, index patients’ pseudonymous email sex partners were less likely to be evaluated (80.7% versus 95.4%, *p*<0.001). Among evaluated partners, there was no significant difference in the proportion of pseudonymous versus control sexual partners that were infected (26.8% versus 29.9%, *p*=0.601).	Low
Woodhouse et al.^48^	In the Colorado Springs population (military and non-military), the percentage of repeat gonococcal infections declined from 10.4% to 8.1% in the period with intensive case-finding (*p*<0.001). The annual incidence of gonorrhea diagnoses declined by 12.9% in the period with intensive case-finding, with the decline primarily found in the civilian population. The 12.9% decline was more pronounced than among the rest of Colorado (6.6% decline) and the US as a whole (2.6% decline); statistical significance not relevant because these are reported cases, not estimates.	Medium

Notes: Reported outcomes are limited to those with statistical comparisons. The strength of evidence summary scores were determined by considering holistically the JBI critical appraisal checklists, study design, and key limitations. The strength of evidence scores should not be interpreted as an assessment of study quality. It is possible for high-quality studies to yield a low strength of evidence for causal inference due to difficulties in measuring the impact of DIS activities and other factors. CDC, 1992^35^ and Engelgau et al., 1995^37^ reported identical findings because they studied the same intervention with similar data.

aRR, adjusted relative risk; CI, confidence interval; DIS, disease intervention specialists; FSU, Field Services Unit; HIV, human immunodeficiency virus; MSM, men who have sex with men; NYC, New York City; OR, odds ratio; PWH, persons with HIV; PrEP, pre-exposure prophylaxis; PPD, purified protein derivative; RR, relative risk; STI, sexually transmitted infection.
