# Supplementary material for: Disease Intervention Specialist-Delivered Interventions and Other Partner Services for HIV and Sexually Transmitted Infections: A Systematic Review
Source: Am J Prev Med. Author manuscript; Available in PMC 2025 Jan 1. (PMC11663095; doi:10.1016/j.amepre.2024.08.004)
Supplement: Appendix [file NIHMS2037260-supplement-Appendix.pdf]

Disease intervention specialist-delivered interventions and other partner services for HIV and sexually transmitted infections: A systematic review

Erika G. Martin, PhD MPH[1,2]; Arzana Myderrizi, MA[2]; Heeun Kim, MA[2]; Patrick Schumacher, MPH[2]; Soyun Jeong, MS[2]; Thomas L. Gift, PhD[3]; Angela B. Hutchinson, PhD MPH[4]; Kevin P. Delaney, PhD[4]; Harrell W. Chesson, PhD[3]

[1] Public Health Accreditation Board, Alexandria, VA

[2] Department of Public Administration and Policy, Rockefeller College of Public Affairs and Policy, University at Albany, Albany, NY

[3] Division of STD Prevention, National Center for HIV, Viral Hepatitis, STD, and TB Prevention, Centers for Disease Control and Prevention, Atlanta, GA

[4] Division of HIV Prevention, National Center for HIV, Viral Hepatitis, STD, and TB Prevention, Centers for Disease Control and Prevention, Atlanta, GA

American Journal of Preventive Medicine

Corresponding author: Erika Martin, Public Health Accreditation Board, 1600 Duke Street, Suite 450, Alexandria, VA 22314; emartin@phaboard.org

These supplemental materials contain the following:

- Full list of search terms used in the keyword search
- Data extraction form
- Completed checklists for the JBI Critical Appraisal tools used in the assessment of the strength of the evidence
- Details on how we scored articles as having low, medium, or high strength of evidence

Full List of Search Terms

Database	Search Terms
PubMed/ MEDLINE	**Initial Search**<br>((“contact tracing” [Title/Abstract]) OR (“disease intervention specialist*” [Title/Abstract]) OR (“partner services” [Title/Abstract]) OR (“partner notification” [Title/Abstract]) OR (“disease investigation specialist*” [Title/Abstract]) OR (“disease intervention staff” [Title/Abstract]) OR (“contact notification” [Title/Abstract]) OR (“communicable disease investigator*” [Title/Abstract]) OR (“field-delivered testing” [Title/Abstract]) OR (“field delivered testing” [Title/Abstract]) OR (“field-delivered treatment” [Title/Abstract]) OR (“field delivered treatment” [Title/Abstract]) OR (“field-delivered therapy” [Title/Abstract]) OR (“field delivered therapy” [Title/Abstract]) OR (“partner counseling and referral services” [Title/Abstract]) OR (“patient-delivered partner therapy” [Title/Abstract]) OR (“patient delivered partner therapy” [Title/Abstract]) OR (“expedited partner therapy” [Title/Abstract]) OR (“treatment verification” [Title/Abstract]) OR (“directly observed therapy” [Title/Abstract])) AND ((STI [Title/Abstract]) OR (“sexually transmitted infection*” [Title/Abstract]) OR (“sexually transmitted disease*” [Title/Abstract]) OR (STD [Title/Abstract]) OR (HIV [Title/Abstract]) OR (“human immunodeficiency virus” [Title/Abstract]) OR (tuberculosis [Title/Abstract]) OR (syphilis [Title/Abstract]) OR (“treponema pallidum” [Title/Abstract]) OR (gonorrhea [Title/Abstract]) OR (“neisseria gonorrhoeae”[Title/Abstract]) OR (chlamydia [Title/Abstract]) OR (“chlamydia trachomatis” [Title/Abstract])) AND (“United States”[Mesh])<br>**Supplemental Search**<br>((“contact investigation” [Title/Abstract]) OR (“venue-based testing” [Title/Abstract]) OR (“venue based testing” [Title/Abstract]) OR (“venue based HIV testing” [Title/Abstract])) AND ((STI [Title/Abstract]) OR (“sexually transmitted infection*” [Title/Abstract]) OR (“sexually transmitted disease*” [Title/Abstract]) OR (STD [Title/Abstract]) OR (HIV [Title/Abstract]) OR (“human immunodeficiency virus” [Title/Abstract]) OR (tuberculosis [Title/Abstract]) OR (syphilis [Title/Abstract]) OR (“treponema pallidum” [Title/Abstract]) OR (gonorrhea [Title/Abstract]) OR (“neisseria gonorrhoeae”[Title/Abstract]) OR (chlamydia [Title/Abstract]) OR (“chlamydia trachomatis” [Title/Abstract])) AND (“United States”[Mesh])<br>**Limits Applied**<br>English language; note that United States region is not included as a limit because it is captured in the MeSH term
Web of Science Core Collection	**Initial Search**<br>(AB = (“contact tracing” OR “disease intervention specialist*” OR “partner services” OR “partner notification” OR “disease investigation specialist*” OR “disease intervention staff” OR “contact notification” OR “communicable disease investigator*” OR “field-delivered testing” OR “field delivered testing” OR “field-delivered treatment” OR “field delivered treatment” OR “field-delivered therapy” OR “field delivered therapy” OR “partner counseling and

Database	Search Terms
	referral services" OR "patient-delivered partner therapy" OR "patient delivered partner therapy" OR "expedited partner therapy" OR "treatment verification" OR "directly observed therapy") AND (AB = (STI OR "sexually transmitted infection*" OR "sexually transmitted disease*" OR STD OR HIV OR "human immunodeficiency virus" OR tuberculosis OR syphilis OR "treponema pallidum" OR gonorrhea OR "neisseria gonorrhoeae" OR chlamydia OR "chlamydia trachomatis")) OR (TI = ("contact tracing" OR "disease intervention specialist*" OR "partner services" OR "partner notification" OR "disease investigation specialist*" OR "disease intervention staff" OR "contact notification" OR "communicable disease investigator*" OR "field-delivered testing" OR "field delivered testing" OR "field-delivered treatment" OR "field delivered treatment" OR "field-delivered therapy" OR "field delivered therapy" OR "partner counseling and referral services" OR "patient-delivered partner therapy" OR "patient delivered partner therapy" OR "expedited partner therapy" OR "treatment verification" OR "directly observed therapy") AND (TI = (STI OR "sexually transmitted infection*" OR "sexually transmitted disease*" OR STD OR HIV OR "human immunodeficiency virus" OR tuberculosis OR syphilis OR "treponema pallidum" OR gonorrhea OR "neisseria gonorrhoeae" OR chlamydia OR "chlamydia trachomatis")))) **<u>Supplemental Search</u>** (AB=("contact investigation" OR "venue-based testing" OR "venue based testing" OR "venue based HIV testing")) AND (AB=(STI OR "sexually transmitted infection*" OR "sexually transmitted disease*" OR STD OR HIV OR "human immunodeficiency virus" OR tuberculosis OR syphilis OR "treponema pallidum" OR gonorrhea OR "neisseria gonorrhoeae" OR chlamydia OR "chlamydia trachomatis")) OR (TI=("contact investigation" OR "venue-based testing" OR "venue based testing" OR "venue based HIV testing")) AND (TI=(STI OR "sexually transmitted infection*" OR "sexually transmitted disease*" OR STD OR HIV OR "human immunodeficiency virus" OR tuberculosis OR syphilis OR "treponema pallidum" OR gonorrhea OR "4eisseria gonorrhoeae" OR chlamydia OR "chlamydia trachomatis")) **<u>Limits Applied</u>** English language; United States region; and articles, review articles, proceeding papers and early access articles
CINAHL	**<u>Initial Search</u>** (((AB ("contact tracing" OR "disease intervention specialist*" OR "partner services" OR "partner notification" OR "disease investigation specialist*" OR "disease intervention staff" OR "contact notification" OR "communicable disease investigator*" OR "field-delivered testing" OR "field delivered testing" OR "field-delivered treatment" OR "field delivered treatment" OR "field-delivered therapy" OR "field delivered therapy" OR "partner counseling and referral services" OR "patient-delivered partner therapy" OR "patient delivered partner therapy" OR "expedited partner therapy" OR "treatment verification" OR "directly observed therapy")) AND ((AB ((STI OR "sexually transmitted infection*" OR "sexually transmitted disease*" OR STD OR HIV OR "human

Database	Search Terms
	immunodeficiency virus" OR tuberculosis OR syphilis OR “treponema pallidum” OR gonorrhea OR "neisseria gonorrhoeae" OR chlamydia OR "chlamydia trachomatis"))) OR (((TI (("contact tracing" OR "disease intervention specialist*" OR "partner services" OR "partner notification" OR "disease investigation specialist*" OR “disease intervention staff” OR “contact notification” OR “communicable disease investigator*” OR “field-delivered testing” OR “field delivered testing” OR “field-delivered treatment” OR “field delivered treatment” OR “field-delivered therapy” OR “field delivered therapy” OR “partner counseling and referral services” OR “patient-delivered partner therapy” OR “patient delivered partner therapy” OR "expedited partner therapy" OR “treatment verification” OR “directly observed therapy”)) AND ((TI ((STI OR "sexually transmitted infection*" OR "sexually transmitted disease*" OR STD OR HIV OR "human immunodeficiency virus" OR tuberculosis OR syphilis OR “treponema pallidum” OR gonorrhea OR "neisseria gonorrhoeae" OR chlamydia OR "chlamydia trachomatis"))) **<u>Supplemental Search</u>**<br>AB ("contact investigation” OR “venue-based testing” OR “venue based testing” OR “venue based HIV testing”))) AND AB (STI OR "sexually transmitted infection*" OR "sexually transmitted disease*" OR STD OR HIV OR "human immunodeficiency virus" OR tuberculosis OR syphilis OR “treponema pallidum” OR gonorrhea OR "neisseria gonorrhoeae" OR chlamydia OR "chlamydia trachomatis") OR TI ("contact investigation” OR “venue-based testing” OR “venue based testing” OR “venue based HIV testing”))) AND TI (STI OR "sexually transmitted infection*" OR "sexually transmitted disease*" OR STD OR HIV OR "human immunodeficiency virus" OR tuberculosis OR syphilis OR “treponema pallidum” OR gonorrhea OR "neisseria gonorrhoeae" OR chlamydia OR "chlamydia trachomatis")<br>**<u>Limits Applied</u>**<br>English language; United States region; and academic journals
ProQuest Dissertations and Theses Global	**<u>Initial Search</u>**<br>(ab("contact tracing" OR "disease intervention specialist*" OR "partner services" OR "partner notification" OR "disease investigation specialist*" OR “disease intervention staff” OR “contact notification” OR “communicable disease investigator*” OR “field-delivered testing” OR “field delivered testing” OR “field-delivered treatment” OR “field delivered treatment” OR “field-delivered therapy” OR “field delivered therapy” OR “partner counseling and referral services” OR “patient-delivered partner therapy” OR “patient delivered partner therapy” OR "expedited partner therapy" OR “treatment verification” OR “directly observed therapy”) AND ab(STI OR "sexually transmitted infection*" OR "sexually transmitted disease*" OR STD OR HIV OR "human immunodeficiency virus" OR tuberculosis OR syphilis OR “treponema pallidum” OR gonorrhea OR "neisseria gonorrhoeae" OR chlamydia OR "chlamydia trachomatis")) OR (ti ("contact tracing" OR "disease intervention specialist*" OR "partner services" OR "partner notification" OR "disease investigation specialist*" OR “disease intervention staff” OR “contact

Database	Search Terms
	notification" OR "communicable disease investigator*" OR "field-delivered testing" OR "field delivered testing" OR "field-delivered treatment" OR "field delivered treatment" OR "field-delivered therapy" OR "field delivered therapy" OR "partner counseling and referral services" OR "patient-delivered partner therapy" OR "patient delivered partner therapy" OR "expedited partner therapy" OR "treatment verification" OR "directly observed therapy") AND ti(STI OR "sexually transmitted infection*" OR "sexually transmitted disease*" OR STD OR HIV OR "human immunodeficiency virus" OR tuberculosis OR syphilis OR "treponema pallidum" OR gonorrhea OR "neisseria gonorrhoeae" OR chlamydia OR "chlamydia trachomatis"))<br>**Supplemental Search**<br>(ab("contact investigation" OR "venue-based testing" OR "venue based testing" OR "venue based HIV testing") AND ab(STI OR "sexually transmitted infection*" OR "sexually transmitted disease*" OR STD OR HIV OR "human immunodeficiency virus" OR tuberculosis OR syphilis OR "treponema pallidum" OR gonorrhea OR "neisseria gonorrhoeae" OR chlamydia OR "chlamydia trachomatis")) OR (ti ("contact investigation" OR "venue-based testing" OR "venue based testing" OR "venue based HIV testing") AND ti(STI OR "sexually transmitted infection*" OR "sexually transmitted disease*" OR STD OR HIV OR "human immunodeficiency virus" OR tuberculosis OR syphilis OR "treponema pallidum" OR gonorrhea OR "neisseria gonorrhoeae" OR chlamydia OR "chlamydia trachomatis"))<br>**Limits Applied**<br>English language; note that it is not feasible to limit to the United States in the search engine. Results will go through two phases of screening. In the first phase, titles and abstracts will be screened to exclude dissertations that are clearly not US region. The remaining titles and abstracts will then be added to the results from the other searches for the title/abstract review for primary research and the PICOTS criteria.

Data Extraction Form

Doc Type Code. Author 1 last name, Author 2 last name, Author 3 last name, year

General Information

Title	
Author	
Journal	
Year	
Date form updated (mm/dd/yyyy)	
Name of Reviewer #1	
Name of Reviewer #2	
Publication type *(journal article, conference abstract, or dissertation)*	
Notes:	

Step 1. Eligibility Screening with Full-Text Review

See the study protocol for details on what is included or excluded in each category. As you work your way down the hierarchy, if you check "no" then all subsequent criteria should be coded as N/A.

**Eligibility Criteria**	**Are eligibility criteria met?**				**Location in text or source** ***(pg., fig/table)***	**Notes on classification**
	**Yes**	**No**	**Unsure**	**N/A**		
Primary data analysis	☐	☐	☐			
Intervention	☐	☐	☐	☐		
Comparator *(Note: must include a statistical test with a CI or p-value to assess statistical significance)*	☐	☐	☐	☐		
Population	☐	☐	☐	☐		
Outcomes	☐	☐	☐	☐		
Final eligibility decision: ☐ INCLUDE ☐ EXCLUDE ☐ UNSURE (team discussion needed)						
Additional information on the comparator: <br>Does this article meet all criteria ***<u>except</u>*** the comparator? (i.e., it is the correct intervention, population, and outcomes but there is no comparator)?						

Eligibility Criteria	Are eligibility criteria met?				Location in text or source *(pg., fig/table)*	Notes on classification
	**Yes**	**No**	**Unsure**	**N/A**		

☐ Yes ☐ No ☐ Unsure ☐ N/A (the article is included)

Are you ***only*** excluding this article because it has no statistical test? (i.e., it meets all other criteria, and there is a pre/post or external comparison group, but this is excluded because there is no test of significance)

☐ Yes ☐ No ☐ Unsure ☐ N/A (the article is included)

If so, is there sufficient information in the tables that a reader could potentially calculate a test of statistical significance?

☐ Yes ☐ No ☐ Unsure ☐ N/A

Does this study include a ***cost analysis***? Are there any reported findings on program costs, economic evaluation, etc.?

☐ Yes ☐ No ☐ Unsure

Notes (optional):

END OF STEP 1 - STOP HERE!

WE WILL PROCEED WITH THE DATA EXTRACTION AFTER A 2ND RESEARCHER REVIEWS THE ASSESSMENT AND OUR TEAM AGREES THAT THE STUDY SHOULD BE INCLUDED.

Step 2. Data Extraction for Included Studies

Note: Record any missing information as "unclear" or "not described."

**Study Characteristics**	**Descriptions as stated in report/paper**	**Location in text or source**
Aim of study as described by the authors. *Copy and paste the direct quote, "in quotations."*<br><br>*You do not need to do any interpretation here – write the authors' aim(s). (They may not all be relevant to our systematic review.)*		
Specific DIS-delivered intervention(s)<br><br>*If there is a describe the "control" or comparison intervention, also describe them here. (Not applicable for pre-and-post designs.)*		
Infection(s)	☐ HIV<br>☐ Chlamydia<br>☐ Gonorrhea<br>☐ Syphilis<br>☐ Tuberculosis<br>☐ Other: ____________________	
Study Design<br><br>*(Randomized design; external control group; pre-and-post design with no external control group; pre-and-post design with external control group – most likely, the paper won't use these terms so use the description from the paper and you can add your own interpretation if relevant)*		

Study Characteristics	Descriptions as stated in report/paper	Location in text or source
Statistical analysis<br><br>*Provide additional details on the statistical analysis performed.*		
Comparator<br><br>*(e.g. partner services vs no partner services, different components of partner services compared to each other, or a new delivery method compared to a traditional approach)*		
Data source<br><br>*(Examples: partner services program data, medical records, patient surveys, surveillance)*		
Start & end date of data		
Start & end date of intervention		
Geographic location<br><br>*(state and city/county; if this is statewide list "statewide" and if this is a specific clinic, please explain)*		
Population<br><br>*Provide a general description (e.g., "MSM") and list specific inclusion or exclusion criteria.*		
Study sample<br><br>*Provide the N (overall & by treatment group). Add comments about attrition, if described.*		

**Study Characteristics**	**Descriptions as stated in report/paper**	**Location in text or source**
Outcome measures<br><br>*Focus on the outcome measures that are used in a statistical analysis.*		
Key findings on outcome measures.<br><br>- *Only include findings with statistical tests of significance.*<br>- *Extract all results related to <u>outcome</u> measures (you do not need to extract findings that are not directly related to the outcomes such as comparison of population characteristics across intervention groups)*<br>- *If there are multiple results such as descriptives, bivariates, and a multivariable regression, extract results from the highest-order analyses like the regression.*<br>- *Please list as bullets, e.g., "partners notified: XX". Include effect sizes and statistical significance.*		

Study Characteristics	Descriptions as stated in report/paper	Location in text or source
If relevant, summarize what results are available without a statistical test (note: you do not need to extract these results, just summarize what is available)<br><br>*Use your best judgment – include here any descriptive findings that are very relevant to the analysis*		
Reported limitations<br><br>*If not describe, write “none listed”*		
Notes (optional):		

Appendix Table 1. JBI Critical Appraisal Checklist for Studies with Quasi-Experimental Designs

Study	Q1. Is it clear what is the "cause" and what is the "effect" (i.e., no confusion about which variable comes first)?	Q2. Were the participants included in any comparisons similar?	Q3. Were the participants included in any comparisons receiving similar treatment, other than the intervention?	Q4. Was there a control group?	Q5. Were there multiple measurements of the outcome both pre and post the intervention?	Q6. Was follow-up complete and if not, were differences between groups in terms of their follow up adequately described and analyzed?	Q7. Were outcomes of participants included in any comparisons measured in the same way?	Q8. Were outcomes measured in a reliable way?	Q9. Was appropriate statistical analysis used?
Billock et al., 2021[1]	Yes	Unclear	Unclear	Yes	No	Unclear	Yes	Unclear	Yes
Bocour et al., 2013[2]	Yes	No	Unclear	Yes	No	Unclear	Yes	Unclear	Yes
CDC, 1992[3]	Yes	Unclear	Unclear	No	Partially	Unclear	Yes	Unclear	No
Du et al., 2007[4]	Yes	Unclear	Unclear	Yes	Yes	Yes	Yes	Unclear	Yes
Engelgau et al., 1995[5]	Yes	Yes	Partially	No	Partially	Unclear	Yes	Unclear	Partially
Halkitis et al., 2011[6]	Yes	No	No	Yes	No	Unclear	Yes	Unclear	Yes
Han et al., 1999[7]	Yes	Unclear	Unclear	Yes	Yes	Not applicable	Yes	Yes	Unclear
Heumann et al., 2017[8]	Yes	No	Unclear	Yes	No	Unclear	Yes	Unclear	Yes
Hood et al., 2017[9]	Yes	Unclear	Unclear	Yes	No	Unclear	Yes	Unclear	Yes
Hoxworth et al. 2003[10]	Yes	No	Yes	Yes	Yes	No	Yes	Unclear	Partially
Katz et al., 1988[11]	Yes	Partially	Unclear	Yes	No	Unclear	Yes	Unclear	Yes
Katz et al., 2016[12]	Unclear	Unclear	Unclear	No	Partially	Unclear	Yes	Unclear	Partially
Malave et al., 2008[13]	Yes	Partially	Yes	Yes	No	Partially	Yes	Unclear	Partially
Renaud et al., 2011[14]	Yes	Unclear	Partially	No	Partially	Unclear	Yes	Unclear	Partially

Ronen et al., 2019[15]	Yes	Unclear	Yes	Yes	Partially	Unclear	Yes	Unclear	Yes
Rosenberg, 1997[16]	Yes	Yes	No	No	Partially	Unclear	Yes	Unclear	No
Steiner et al., 2003[17]	Yes	Unclear	Unclear	No	Partially	Unclear	Yes	Unclear	Partially
Taylor et al., 2010[18]	Yes	Partially	Unclear	No	Partially	Unclear	Yes	Unclear	Yes
Tributino et al., 2018[19]	Yes	Yes	Unclear	No	Partially	Unclear	Yes	Unclear	Partially
Udeagu et al., 2012[20]	Yes	No	Unclear	Yes	Partially	Unclear	Yes	Unclear	Partially
Udeagu et al., 2014[21]	Yes	Partially	Unclear	Yes	No	No	Yes	Unclear	Yes
Udeagu et al., 2014[22]	Yes	No	Unclear	Yes	Yes	Unclear	Yes	Unclear	Partially
Vest et al., 2007[23]	Yes	No	Unclear	Yes	No	Unclear	Yes	Unclear	Partially
Woodhouse et al., 1986[24]	Yes	Unclear	Unclear	Partially	Yes	Unclear	Yes	Unclear	Partially

Appendix Table 2. JBI Critical Appraisal Checklist for Studies with Randomized Controlled Trial Designs

Study	Q1. Was true random-ization used for assign-ment of participants to treatment groups?	Q2. Was allocation to groups concealed?	Q3. Were treatment groups similar at baseline?	Q4. Were participants blind to treatment assign-ment?	Q5. Were those delivering treatment blind to treatment assign-ment?	Q6. Were outcome assessors blind to treatment assign-ment?	Q7. Were treatment groups treated identically other than the intervention of interest?	Q8. Was follow up complete and if not, were differences between groups in terms of their follow up adequately described and analyzed?	Q9. Were participants analyzed in the groups to which they were random-ized?	Q10. Were outcomes measured in the same way for treatment groups?	Q11. Were outcomes measured in a reliable way?	Q12. Was appro-priate stat-istical analysis used?	Q13. Was the trial design appro-priate and any deviations from the standard RCT design accounted for in the conduct and analysi of the trial?
Brewer et al., 2005[25]	Yes	Unclear	Unclear	Unclear	Unclear	Unclear	Yes	Unclear	Yes	Yes	Unclear	Partially	Yes
Golden et al., 2015[26]	Yes	Yes	Partially	No	Yes	No	Partially	Unclear	Unclear	Yes	Unclear	Yes	Yes
Kerani et al., 2011[27]	Yes	Unclear	Partially	Unclear	Unclear	Unclear	Yes	No	Partially	Yes	Unclear	Partially	Yes
Landis et al., 1992[28]	Unclear	Unclear	Unclear	Unclear	Unclear	Unclear	Yes	Unclear	Yes	Yes	Unclear	Partially	Yes
Schwebke & Desmond, 2010[29]	Unclear	Unclear	Yes	Unclear	Unclear	Unclear	Unclear	Yes	Yes	Yes	Yes	Partially[1]	Yes

[1] Appropriate statistical analysis was used but there was an error in the calculation of the main finding.

Appendix Table 3. JBI Critical Appraisal Checklist for Studies with Cross-Sectional Designs

Study	Q1. Were the criteria for inclusion in the sample clearly defined?	Q2. Were the study subjects and the setting described in detail?	Q3. Was the exposure measured in a reliable way?	Q4. Were objective, standard criteria used for measurement of the condition?	Q5. Were confounding factors identified?	Q6. Were strategies to deal with confounding factors stated?	Q7. Were the outcomes measured in a valid and reliable way?	Q8. Was appropriate statistical analysis used?
Golden et al., 2009[30]	Yes	Yes	Unclear	No	Partially	Yes	Unclear	Yes

Details on how articles were scored on strength of evidence

The appraisal was based on a totality of considerations based on the completed JBI critical appraisal checklists, the study design, and key limitations. To complete the checklists, one reviewer first scored each item with narrative comments explaining their scores on each component. The reviewer then made a judgment of low quality, medium quality, or high quality of evidence for causal inference based on a reflection of the component scores, the study design, and study limitations that were identified by either the study authors or the reviewer. For example, one principle is that a well-executed randomized controlled trial would be "high" quality evidence and a well-executed pre-post comparative study would have been "medium" quality evidence. Yet if an observational study had implemented a widely-used quantitative causal inference technique such as a synthetic matched control or a differences-in-differences regression design for pre-post comparisons between groups, reviewers would have considered increasing the quality of evidence to "high." Evidence was downgraded if there were one more critical weaknesses such as insufficient sample sizes, not adjusting for nonequivalence between groups, treatment spillover, or incorrect calculations in the results tables, et cetera. The reviewer added narrative comments to the internal scoring rubric to explain their reasoning for the overall score.

Ultimately, these scores require judgment, and several procedures were implemented to ensure consistency in coding. First, a second reviewer verified all JBI checklists scores and the overall score and wrote additional comments regarding their interpretations. At least one additional author reviewed the article in instances where there was disagreement about the scores. Second, articles were discussed during weekly meetings for ongoing calibration of scores across reviewers. In several instances, this resulted in going back to earlier articles to revise scores for consistency across articles.

Although the JBI critical appraisal checklists allowed for a detail review of different dimensions of each article, a challenge with checklists is that they often capture the quality of reporting and not the quality of the research design and execution. If an article scores well on most dimensions but has one "fatal" weakness, that may be missed by simply summing scores from the checklist to create numerical thresholds. A final consideration for the holistic approach used in this review is that many articles did not adhere to common reporting guidelines such as the STROBE Statement. Consequently, many articles had JBI critical appraisal component items rated as "unsure," which does not reflect the quality of the evidence.

References

1. Billock RM, Samoff E, Lund JL, Pence BW, Powers KA. HIV viral suppression and pre-exposure prophylaxis in HIV and syphilis contact tracing networks: An analysis of disease surveillance and prescription claims data. *J Acquir Immune Defic Syndr*. 2021;88(2):157-164. doi:10.1097/QAI.0000000000002739

2. Bocour A, Renaud TC, Udeagu CCN, Shepard CW. HIV partner services are associated with timely linkage to HIV medical care. *AIDS*. 2013;27(18):2961-2963. doi:10.1097/QAD.0000000000000031

3. Centers for Disease Control (CDC). Epidemic early syphilis-Montgomery County, Alabama, 1990-1991. *MMWR Morb Mortal Wkly Rep*. 1992;41(42):790-794.

4. Du P, Coles FB, Gerber T, McNutt LA. Effects of partner notification on reducing gonorrhea incidence rate. *Sex Transm Dis*. 2007;34(4):189-194. doi:10.1097/01.olq.0000237861.47751.16

5. Engelgau MM, Woernle CH, Rolfs RT, Greenspan JR, O'Cain M, Gorsky RD. Control of epidemic early syphilis: The results of an intervention using social networks. *Sex Transm Dis*. 1995;22(4):203-209.

6. Halkitis PN, Kupprat SA, McCree DH, et al. Evaluation of the relative effectiveness of three HIV testing strategies targeting African American men who have sex with men (MSM) in New York City. *Ann Behav Med*. 2011;42(3):361-369. doi:10.1007/s12160-011-9299-4

7. Han Y, Coles FB, Muse A, Hipp S. Assessment of a geographically targeted field intervention on gonorrhea incidence in two New York State counties. *Sex Transm Dis*. 1999;26(5):296-302. doi:10.1097/00007435-199905000-00011

8. Heumann CL, Katz DA, Dombrowski JC, Bennett AB, Manhart LE, Golden MR. Comparison of in-person versus telephone interviews for early syphilis and HIV partner services in King County, Washington (2010-2014). *Sex Transm Dis*. 2017;44(4):249-254. doi:10.1097/OLQ.0000000000000583

9. Hood JE, Katz DA, Bennett AB, et al. Integrating HIV surveillance and field services: Data quality and care continuum in King County, Washington, 2010-2015. *Am J Public Health*. 2017;107(12):1938-1943. doi:10.2105/AJPH.2017.304069

10. Hoxworth T, Spencer NE, Peterman TA, Craig T, Johnson S, Maher JE. Changes in partnerships and HIV risk behaviors after partner notification. *Sex Transm Dis*. 2003;30(1):83-88. doi:10.1097/00007435-200301000-00016

11. Katz BP, Danos CS, Quinn TS, Caine V, Jones RB. Efficiency and cost-effectiveness of field follow-up for patients with Chlamydia trachomatis infection in a sexually transmitted diseases clinic. *Sex Transm Dis*. 1988;15(1):11-16. doi:10.1097/00007435-198801000-00003

12. Katz DA, Dombrowski JC, Kerani RP, et al. Integrating HIV testing as an outcome of STD partner services for men who have sex with men. *AIDS Patient Care STDS*. 2016;30(5):208-214. doi:10.1089/apc.2016.0027

13. Malave MC, Shah D, Sackoff JE, Rubin S, Begier EM. Human immunodeficiency virus partner elicitation and notification in New York City: Public health does it better. *Sex Transm Dis*. 2008;35(10):869-876. doi:10.1097/OLQ.0b013e31817d2f82

14. Renaud TC, Wong MR, Bocour A, et al. The effect of HIV field-based testing on the proportion of notified partners who test for HIV in New York City. *Am J Public Health*. 2011;101(7):1168-1171. doi:10.2105/AJPH.2011.300129

15. Ronen K, Golden MR, Dombrowski JC, Kerani RP, Bell TR, Katz DA. Uptake and impact of short message service reminders via sexually transmitted infection partner services on human immunodeficiency virus/sexually transmitted infection testing frequency among men who have sex with men. *Sex Transm Dis*. 2019;46(10):641-647. doi:10.1097/OLQ.0000000000001043

16. Rosenberg DM. *The Utility of Social Network Methods for Identifying Persons with and at Risk for Sexually Acquired Syphilis*. Tulane University; 1997.

17. Steiner KC, Davila V, Kent CK, Chaw JK, Fischer L, Klausner JD. Field-delivered therapy increases treatment for chlamydia and gonorrhea. *Am J Public Health*. 2003;93(6):882-884. doi:10.2105/ajph.93.6.882

18. Taylor MM, Mickey T, Winscott M, James H, Kenney K, England B. Improving partner services by embedding disease intervention specialists in HIV-clinics. *Sex Transm Dis*. 2010;37(12):767-770. doi:10.1097/OLQ.0b013e3181e65e8b

19. Tributino A, Montgomery MC, Bertrand T, et al. Partner notification outcomes after integration of an on-site disease intervention specialist at a sexually transmitted disease clinic. *PLoS One*. 2018;13(3):e0194041. doi:10.1371/journal.pone.0194041

20. Udeagu CCN, Shah D, Shepard CW, Bocour A, Guiterrez R, Begier EM. Impact of a New York City health department initiative to expand HIV partner services outside STD clinics. *Public Health Rep*. 2012;127(1):107-114.

21. Udeagu CCN, Bocour A, Shah S, Ramos Y, Gutierrez R, Shepard CW. Bringing HIV partner services into the age of social media and mobile creativity. *Sex Transm Dis*. 2014;41(10):631-636. doi:10.1097/OLQ.0000000000000181

22. Udeagu CCN, Bocour A, Shepard CW. For partner services, do we need “face-time,” or FaceTime? Trends in relative proportion of in-person notifications and HIV testing after introduction of a telephone option for HIV exposure notification by public health. *Sex Transm Dis*. 2014;41(11):671-673. doi:10.1097/OLQ.0000000000000192

23. Vest JR, Valadez AM, Hanner A, Lee JH, Harris PB. Using e-mail to notify pseudonymous e-mail sexual partners. *Sex Transm Dis*. 2007;34(11):840-845. doi:10.1097/OLQ.0b013e318073bd5d

24. Woodhouse DE, Potterat JJ, Muth JB, Pratts CI, Rothenberg RB, Fogle JS. A civilian-military partnership to reduce the incidence of gonorrhea. *Public Health Rep*. 1985;100(1):61-65.

25. Brewer DD, Potterat JJ, Muth SQ, et al. Randomized trial of supplementary interviewing techniques to enhance recall of sexual partners in contact interviews. *Sex Transm Dis*. 2005;32(3):189-193. doi:10.1097/01.olq.0000153574.38764.0e

26. Golden MR, Kerani RP, Stenger M, et al. Uptake and population-level impact of expedited partner therapy (EPT) on Chlamydia trachomitis and Neisseria gonorrhoeae: The Washington State community-level randomized trial of EPT. *PLoS Med*. 2015;12(1):e1001777. doi:10.1371/journal.pmed.1001777

27. Kerani RP, Fleming M, DeYoung B, Golden MR. A randomized, controlled trial of inSPOT and patient-delivered partner therapy for gonorrhea and chlamydial infection among men who have sex with men. *Sex Transm Dis*. 2011;38(10):941-946. doi:10.1097/OLQ.0b013e318223fcbc

28. Landis SE, Schoenbach VJ, Weber DJ, et al. Results of a randomized trial of partner notification in cases of HIV infection in North Carolina. *N Engl J Med*. 1992;326(2):101-106. doi:10.1056/NEJM199201093260205

29. Schwebke JR, Desmond RA. A randomized controlled trial of partner notification methods for prevention of trichomoniasis in women. *Sex Transm Dis*. 2010;37(6):392-396. doi:10.1097/OLQ.0b013e3181dd1691

30. Golden MR, Dombrowski JC, Wood RW, Fleming M, Harrington RD. A controlled study of the effectiveness of public health HIV partner notification services. *AIDS*. 2009;23(1):133-138. doi:10.1097/QAD.0b013e32831fb52f
